# Paradoxical Response to Neoadjuvant Therapy in Undifferentiated Pleomorphic Sarcoma: Increased Tumor Size on MRI Associated with Favorable Pathology

**DOI:** 10.3390/cancers17050830

**Published:** 2025-02-27

**Authors:** Mariam H. Goreish, Nicolò Gennaro, Laetitia Perronne, Gorkem Durak, Amir A. Borhani, Hatice Savas, Linda Kelahan, Ryan Avery, Kamal Subedi, Tugce Agirlar Trabzonlu, Ulas Bagci, Baris Turkbey, Spyridon Bakas, Sean Sachdev, Ronen Sumagin, Borislav A. Alexiev, Pedro Hermida de Viveiros, Seth M. Pollack, Yuri S. Velichko

**Affiliations:** 1 Department of Radiology, Feinberg School of Medicine, Northwestern University, Chicago, IL 60611, USAlaetitia.perronne@northwestern.edu (L.P.); gorkem.durak@northwestern.edu (G.D.); amir.borhani@nm.org (A.A.B.); ryan.avery@nm.org (R.A.);; 2 Molecular Imaging Branch, National Cancer Institute, National Institutes of Health, Bethesda, MD 20892, USA; 3 Department of Pathology, School of Medicine, Indiana University, Indianapolis, IN 46202, USA; 4 Department of Radiation Oncology, Feinberg School of Medicine, Northwestern University, Chicago, IL 60611, USA; 5 Robert H. Lurie Comprehensive Cancer Center, Feinberg School of Medicine, Northwestern University, Chicago, IL 60611, USA; 6 Department of Pathology, Feinberg School of Medicine, Northwestern University, Chicago, IL 60611, USA; ronen.sumagin@northwestern.edu; 7 Division of Hematology and Oncology, Feinberg School of Medicine, Northwestern University, Chicago, IL 60611, USA

**Keywords:** sarcoma, neoadjuvant chemoradiotherapy, MRI, RECIST, pathology

## Abstract

**Background/Objectives**: To correlate size changes in undifferentiated pleomorphic sarcoma (UPS) on magnetic resonance imaging (MRI) after neoadjuvant chemoradiation therapy (nCRT) with pathological response, risk of local recurrence, and therapeutic regimens. **Methods**: This retrospective study analyzed clinical, pathological, and imaging data from 39 biopsy-proven UPS subjects. Four readers measured the tumor dimensions before and after nCRT, including two perpendicular axial diameters and the longest coronal/sagittal diameter. Three cross-sectional areas and bounding volume were also calculated. Responders (pR) were defined as having ≤10% viable cells and non-responders (pNR) as having more. Inter-reader agreement was evaluated using Kendall’s concordance coefficient. Changes in tumor size were compared between pR and pNR using one-way ANOVA and Tukey’s HSD test for multiple comparisons of means. **Results**: pR showed a greater increase in size across all measurements compared to pNR. For the longest axial diameter, the mean increase was 30% ± 35% for pR and 14% ± 31% for pNR, with a mean difference (pR-pNR) of 16% (95% CI: 6–27%, *p* = 0.003). In tumors treated with radiotherapy alone, pR exhibited larger size increases in all dimensions compared to pNR. In contrast, in the chemoradiation group, pR showed a slight increase, while pNR generally shrank, although these differences did not reach statistical significance. Notably, pNR with local recurrence exhibited a reduction in all tumor dimensions compared to pNR without local recurrence. **Conclusions**: This exploratory study suggests that tumor size changes may predict pathological response and local recurrence after nCRT in UPS; however, the small sample size limits the generalizability of these findings.

## Simple Summary

MRI measurements can effectively differentiate UPS patients into good and poor pathological responders to neoadjuvant therapy. Interestingly, a tumor size increase across all dimensions on MRI may paradoxically indicate a favorable response. In contrast, tumors exhibiting minimal size increase or even shrinkage on MRI after therapy warrant caution, as they are associated with a higher risk of local recurrence following surgery. These findings highlight the limitations of RECIST criteria in accurately assessing the response of UPS to neoadjuvant therapy.

## 1. Introduction

Sarcomas are a rare and heterogeneous type of cancer that affects the bones, muscles, and connective tissues and accounts for less than 1% of all cancer diagnoses [1,2]. Soft tissue sarcomas (STSs) represent over 77% of all sarcomas [3]. Within STSs, undifferentiated pleomorphic sarcoma (UPS) is the most prevalent subtype. Known for its aggressive behavior and high risk of local recurrence, UPS presents significant challenges for clinical management, particularly in developing and evaluating effective treatment strategies. Limited patient populations make it difficult to conduct large-scale clinical research, highlighting the urgent need for focused studies on rare cancers [4].

Neoadjuvant therapies have emerged as a promising pre-operative strategy for improving surgical outcomes and local tumor control [5,6,7]. The increasing use of neoadjuvant chemoradiotherapy in STSs [8] has revealed that STSs do not always shrink but can instead increase in size. This observation led several studies to investigate the relationship between treatment-induced size changes and tumor response or clinical outcome. However, most of these studies analyzed heterogeneous datasets to overcome data scarcity, leading to inconclusive results. Starting in 2010, Miki et al. observed in a retrospective study of 91 patients (50% UPS) that a size increase of 10% or more did not impact outcomes [9]. Delisca et al. similarly reported in a retrospective study on 70 patients (49% UPS) that a 20% increase in tumor diameter was not associated with worse outcomes compared to those with stable or shrinking tumors [10]. However, Tsagozis et al. demonstrated that volume reduction was a better prognostic indicator than size increase in a mixed group of high- and low-grade STSs [11]. More recently, De Lamarliere et al. [12] highlighted the variability in size changes after nCRT in a small cohort of 13 patients (54% UPS), suggesting the need for treatment adaptations to optimize tumor coverage in UPS. Notably, none of these studies performed subanalysis for specific STS subtypes, despite their distinct biological and clinical characteristics.

This variability in STS response is attributed to treatment-induced effects like hemorrhage, necrosis, or cystic degeneration [13]. Consequently, RECIST (Response Evaluation Criteria in Solid Tumors) [14] may inaccurately assess response due to its reliance on tumor size reduction [15,16,17]. Recognizing this limitation, the European Organization for Research and Treatment of Cancer (EORTC)—Soft Tissue and Bone Sarcoma Group recommends excluding size and volume measurements, except for myxoid liposarcomas, when evaluating response to radiotherapy in clinical practice [18,19,20,21]. To address RECIST’s limitations, the Choi criteria, originally developed for gastrointestinal stromal tumors, have been adapted for MRI to improve STS response assessment by considering changes in both tumor size and enhancement. However, challenges in quantitative MRI analysis and low reproducibility limit the widespread use of the Choi criteria [22,23,24,25].

In managing patients with soft tissue sarcoma, histological analysis from a biopsy remains the gold standard for both diagnosis and treatment planning [26]. It provides the most detailed description of the tumor and highly accurate diagnoses [27,28,29]. However, its use in assessing treatment response is limited by the absence of pathology before surgery. In contrast, imaging offers advantages such as minimal invasiveness, rapid results, and broad visualization, making it the preferred method for evaluating treatment response. MRI is particularly valuable in this context. However, the lack of a reliable, non-invasive method for assessing STS response after nCRT limits clinical decision-making.

In this study, we compare tumor size changes between patients classified as pathological responders versus non-responders, those who experience recurrence versus those who achieve effective local control, and across different neoadjuvant chemo-radiotherapy regimens. By analyzing these relationships, we aim to identify whether MRI-based tumor size changes provide a reliable indicator of treatment response in UPS patients treated with nCRT.

## 2. Materials and Methods

### 2.1. Patient Population

**Figure 1:**
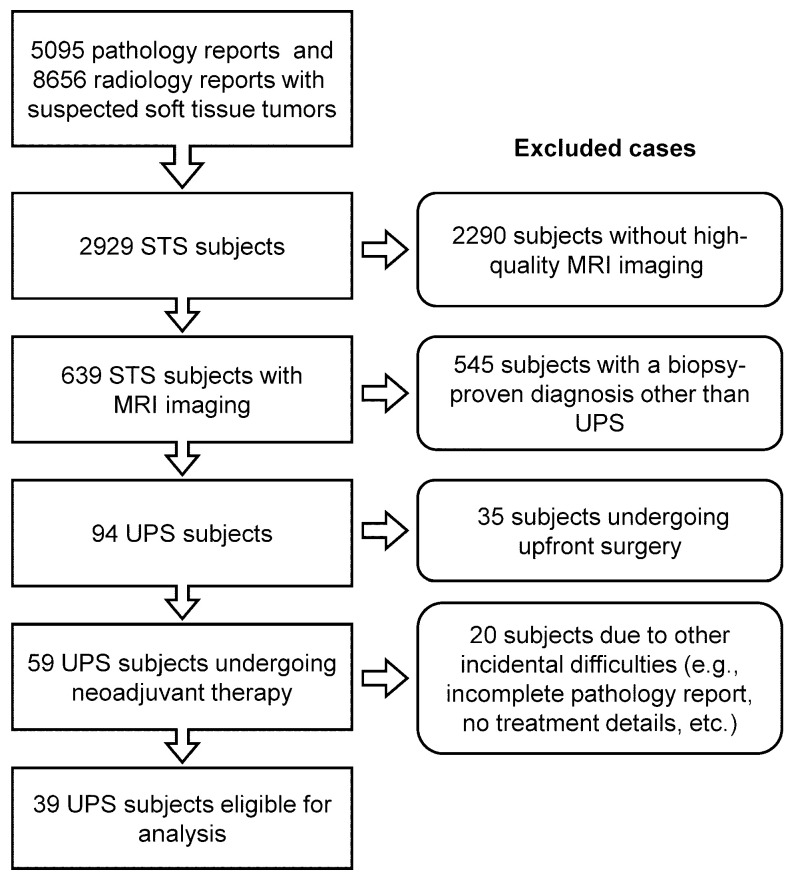
Flowchart for UPS subject inclusion and exclusion.

This retrospective study was approved by the Institutional Review Board (IRB) and received waivers for both Health Insurance Portability and Accountability Act (HIPAA) authorization and written informed consent. The institutional electronic data warehouse (EDW) was searched from January 2004 to December 2022 to identify patients (Figure 1) meeting the following inclusion criteria: diagnosis of biopsy-proven UPS, receipt of neoadjuvant radiotherapy or chemoradiotherapy, pre- and post-treatment MRI of the primary tumor, and surgical pathology analysis of viable and non-viable tumor components (necrotic material or hyalinization tissue). Patient demographics and clinical characteristics are summarized in Table 1, while treatment details and response to therapy are summarized in Table 2.

### 2.2. MRI Imaging Analysis

Both 1.5 and 3T MRI scans from multiple hospitals within a single healthcare system were included. To minimize variability due to scanner differences, all measurements were obtained only from native high-resolution scans in axial, sagittal, or coronal planes. Post-processed reconstructions were excluded from the analysis to avoid potential errors in the measurements. Four readers independently measured all tumors, blinded to histopathology. These readers included three senior radiologists with more than 5 years of experience in body imaging, musculoskeletal imaging, and general diagnostic radiology, respectively, and a fourth-year medical student with 6 months of sarcoma research experience.

Tumor size measurements were performed using contrast-enhanced T1-weighted images before and after treatment, with optional use of fat-saturated T2-weighted images or other sequences (STIR, fat-saturated proton density, etc.) for improved margin definition and measurement accuracy. Two axial diameters included the longest diameter (X), corresponding to the RECIST diameter, and a diameter perpendicular to X (labeled as Y). The third, the longest vertical diameter (Z), was measured on a plane perpendicular to the axial plane. From these, three cross-sectional areas (XY, XZ, and YZ) and the volume (XYZ) were estimated by computing a product of corresponding diameters. Percentage change in size was determined based on pre- and post-treatment measurements. Tumor measurements were performed on the solid tumor mass, excluding infiltrative extensions into surrounding tissues, such as the tail sign.

### 2.3. Histopathology

**Figure 2:**
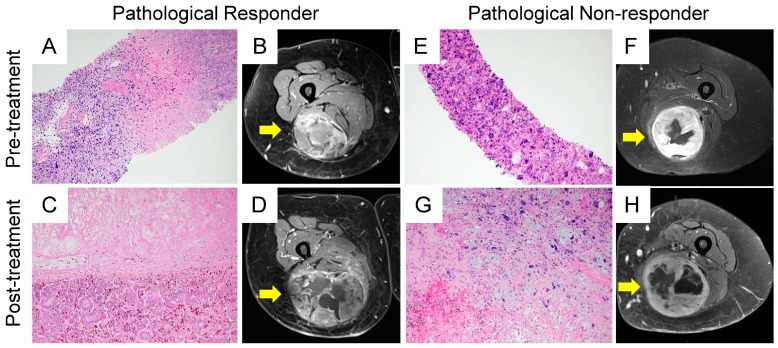
A 77-year-old woman with marked treatment response (pR), diagnosed with UPS with FNCLCC Grade III. (**A**) Pre-treatment core biopsy. The H&E-stained pathology slide shows abundant viable tumor cells colored dark purple (×100 magnification). (**B**) Contrast-enhanced fat-saturated T1-w MRI shows a heterogeneous, enhancing mass deep-seated in the right posterior compartment of the thigh. (**C**) Post-radiotherapy (5000 cGy/25 fractions) resection specimen. H&E-stained pathology slide (×40 magnification) shows tumor bed with marked treatment effect and 0% viable tumor. (**D**) Contrast-enhanced fat-saturated T1-w MRI showed a moderate increase in size compared to baseline and large, central areas of non-enhancing tissue, likely necrotic or hemorrhagic areas. Increase in maximum tumor diameter was +43%, +26%, and +21% in X, Y, and Z directions. (**E**) A 65-year-old woman with mild treatment response (pNR). Pre-treatment core biopsy. H&E pathology slide (×40 magnification) shows a spindle cell and pleomorphic cell population, diagnostic of UPS (Grade II). (**F**) Contrast-enhanced fat-saturated T1-w MRI shows a round-shaped deep-seated mass in the right posterior thigh, with small central areas of non-enhancing tissue. (**G**) Post-radiotherapy (5000 cGy/25 fractions) resection specimen. Tumor area with focal treatment effect (necrosis, chronic inflammation, and fibrosis), approximately 60% viable tumor. Hematoxylin–eosin stain, (×100 magnification). (**H**) Contrast-enhanced fat-saturated T1-w MRI showed a moderate increase in size compared to baseline. Increase in maximum tumor diameter was +41%, +23%, and +15% in X, Y, and Z.

The percentage of viable/stainable tumor cells was collected from histopathology reports. Patients were classified as pathological responders (pR) or non-responders (pNR) based on a 10% viable cell cutoff, as established in previous studies [16,17]. Figure 2 illustrate both pR and pNR cases, demonstrating histopathology slides highlighting viable tumor cells in hematoxylin–eosin (H&E) stained tissue and corresponding MRI images.

### 2.4. Local Recurrence

Local recurrence was defined as a UPS growing in the same or proximate location to the original cancer. Information about local recurrence was obtained from patients’ clinical records at all available time points. All cases of local recurrence, identified initially on imaging or physical examination, were then confirmed through biopsy or surgical pathology.

### 2.5. Statistical Analysis

Power analysis using a two-sample *t*-test was conducted to assess the study’s ability to detect differences in pathological response [30].

Inter-reader agreement for tumor diameter measurements was assessed using Kendall’s W concordance coefficient [31]. To investigate potential differences in tumor measurements, patients were grouped by pathological response (pR vs. pNR), recurrence (with vs. without), and treatment type (radiotherapy vs. chemoradiotherapy). Descriptive statistics (mean, standard deviation (SD), median, and interquartile range (IQR)) for tumor size changes from all four readers were calculated within each group. One-way ANOVA was conducted to compare tumor size changes across these groups, followed by Tukey’s Honestly Significant Difference (HSD) test for pairwise comparisons. Given the small sample size, we explored a fully Bayesian approach as an alternative to ANOVA and Tukey’s HSD tests. This approach allowed for estimation of tumor size changes per group while accounting for inter-reader variability using generalized linear mixed-effects models (GLMMs) with readers as a random effect [32].

To determine the optimal cutoff for tumor size changes that differentiate between pR and pNR, as well as patients with and without recurrence, we used the product of sensitivity and specificity, in analogy to the Youden index [33]. The cutoff value maximizing the product was selected as the optimal cutoff. This metric demonstrates the optimal performance and a good balance between sensitivity and specificity among various models [34]. Given the small sample size, we conducted bootstrapping with 1000 resamples to assess cutoff stability, selecting the cutoff maximizing the product in most bootstrap samples. To compare the diagnostic performance of RECIST 1.1 criteria with criteria based on the optimal tumor size cutoff, we constructed contingency matrices for each. These matrices summarized the agreement between each set of criteria and pathological responses and were used to calculate performance metrics for each set.

Survival analysis was performed using Kaplan–Meier curves to estimate overall survival (OS) and recurrence-free survival (RFS) [35]. Differences between groups were assessed using Cox proportional hazards models [36]. The data were censored at the last follow-up for patients who did not experience the event of interest. All statistical analyses were performed using the statistical environment R version 4.2.2 (Vienna, Austria) [37], with a *p*-value threshold of 0.05 determining statistical significance. Gemini AI (ver. 2.0), developed by Google, was utilized to refine the language and improve clarity within the manuscript.

## 3. Results

### 3.1. Patient Characteristics

Out of the 2929 patients with STSs identified in EDW search, only 39 met the eligibility criteria for analysis (Figure 1), including 23 females (59%) and 16 males (41%). Table 1 provides a detailed overview of their demographics and tumor characteristics. Patients received standardized treatment regimens consisting of either radiotherapy alone or chemoradiotherapy combining radiotherapy with anthracycline-based chemotherapy. The average time between post-treatment and pre-treatment imaging was 89.7 ± 26.1 days (median: 91 days, interquartile range: 70–108 days). The average interval between post-treatment imaging and surgery was 53.7 ± 87.2 days (median: 21 days, interquartile range: 17–36 days). Finally, the average time from surgery to local recurrence was 3.67 ± 3.9 years (median: 2.45 years, interquartile range: 1.3–4.2 years).

Pathological response (pR) was favorable for fifteen patients (38%), including seven females and eight males. Conversely, twenty-four patients (62%) did not respond to treatment, including sixteen females and eight males. Local recurrence occurred in 12 patients (31%), with females being more likely to experience recurrence compared to males (n = 9, 75%). While the local recurrence rate was evenly distributed across tumor grades, three of these cases (25%) had microscopic positive margins managed conservatively before recurrence.

Average tumor diameters obtained from pretreatment scans were 82.4 ± 37.5 mm for X, 66.7 ± 36.6 mm for Y, and 121.5 ± 72.2 mm for Z, respectively, with corresponding median (interquartile range) values of 78.7 mm (62.5, 99.1), 61.9 mm (46, 76.7), and 100.3 mm (77.5, 140.8). All four readers showed strong agreement in estimating the percent change across all tumor sizes. Kendall’s W concordance coefficient exceeded 0.85 for all measures, with corresponding *p*-values below 1 × 10^−12^, indicating highly significant agreement. Detailed results are presented in Table 3.

#### 3.1.1. Power Analysis

As an exploratory, single-institution study with a small sample size (n = 39), this analysis has limited statistical power. Power analysis indicated a power below 0.4 to detect a medium effect size for both pathological response and local recurrence, highlighting the potential for Type II errors. Therefore, these findings should be considered preliminary, and further research with larger, multi-institutional cohorts is necessary to confirm these observations and draw more definitive conclusions.

#### 3.1.2. Correlating Pathological Response with Changes in MRI

**Figure 3:**
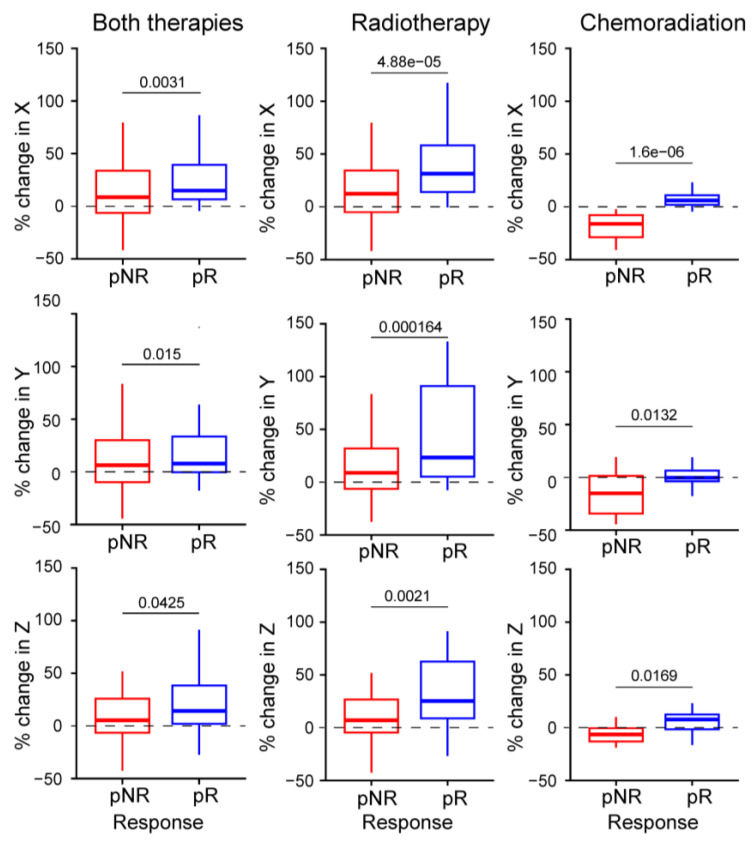
Percentage change in tumor sizes across the main axes (X, Y, and Z) for both therapies and within each therapeutic subgroup, including radiotherapy and chemoradiation, for responders (pR) and non-responders (pNR). Each plot shows a *p*-value for the mean difference (pR-pNR) between responders and non-responders, calculated using Tukey’s HSD test.

Figure 2 shows pre- and post-treatment MRI scans of tumors, illustrating size changes in both responders and non-responders to treatment. Notably, 62% of patients demonstrated increases in both X and Y diameters, while 72% showed an increase in the Z diameter. Responders exhibited a significantly greater increase in tumor size across all measurements compared to non-responders (Table 3). For example, the longest diameter (X) increased by an average of 30% (SD 35%) in responders, compared to 14% (SD 31%) in non-responders, resulting in a considerable difference of 16% (95% CI: 6% to 27%, *p*-value = 0.003). Similarly, the change in cross-sectional area on the axial plane showed a significant difference. Responders experienced a larger average increase (77%, SD 112%) compared to non-responders (36%, SD 75%), with a difference of 41% (95% CI: 12% to 71%, *p* = 0.006). Figure 3 illustrates the changes in tumor size across all main axes for both responders and non-responders. Due to the small sample size, the statistical power of subgroup analyses is limited, and these preliminary results should be interpreted cautiously.

Additionally, Figures S1–S3 (Supplemental Materials) present estimated mean values for each group using the GLMM approach. It displays the median and 90% uncertainty interval, representing the range of possible values for the true mean with a 90% probability. Interestingly, both ANOVA and GLMM analyses yielded nearly identical mean values for all measurements.

### 3.2. Effect of Therapeutic Regimens

To compare the impact of different therapeutic regimens on tumor response, we analyzed radiotherapy and chemoradiation data separately. Tumor size changes in the main diameters for each treatment group are illustrated in Figure 3. Figure S1 further provides a comparative visualization of tumor size changes using a GLMMs approach, demonstrating distinct response patterns between pR and pNR in both treatment groups. Tukey’s HSD test was used to compare responder (pR) and non-responder (pNR) groups within each treatment. The results of this analysis for all measurements are summarized in Table 4. The limited statistical power of each subgroup analysis warrants cautious interpretation of these results.

### 3.3. Local Recurrence Analysis

**Figure 4:**
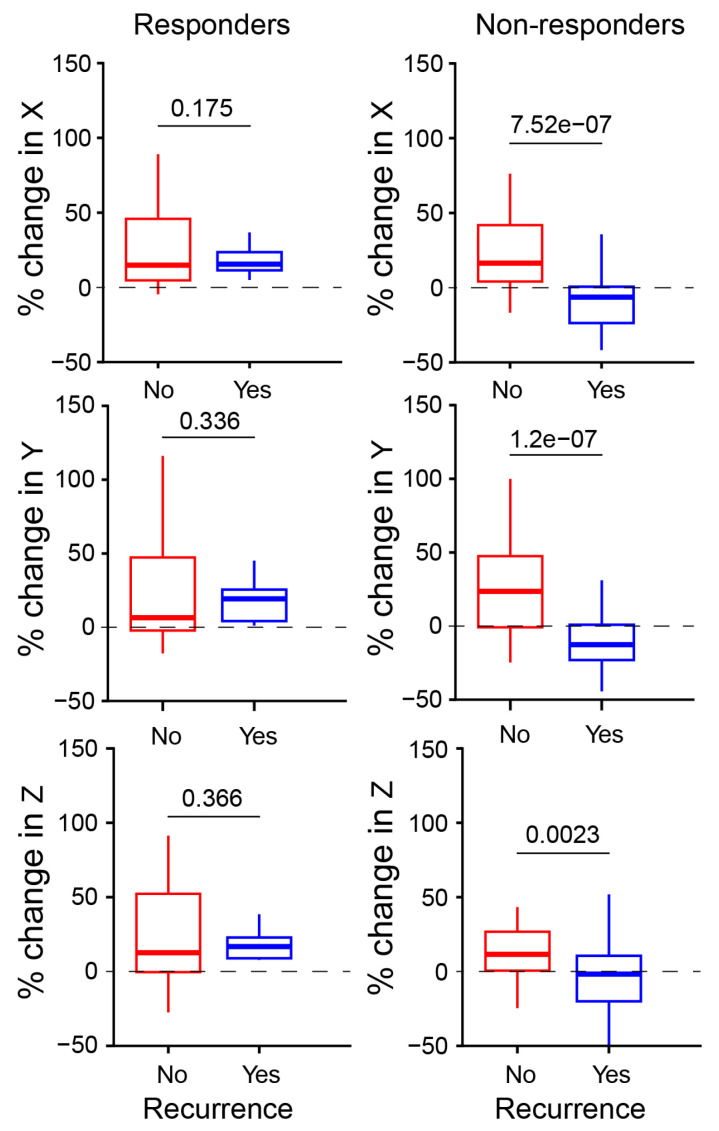
Percentage change in tumor sizes across the main axes (X, Y, and Z) for responders and non-responders, comparing patients with and without recurrence. Each plot shows a p-value for the mean difference between patients with (Yes) and without (No) recurrence, calculated using Tukey’s HSD test.

**Figure 5:**
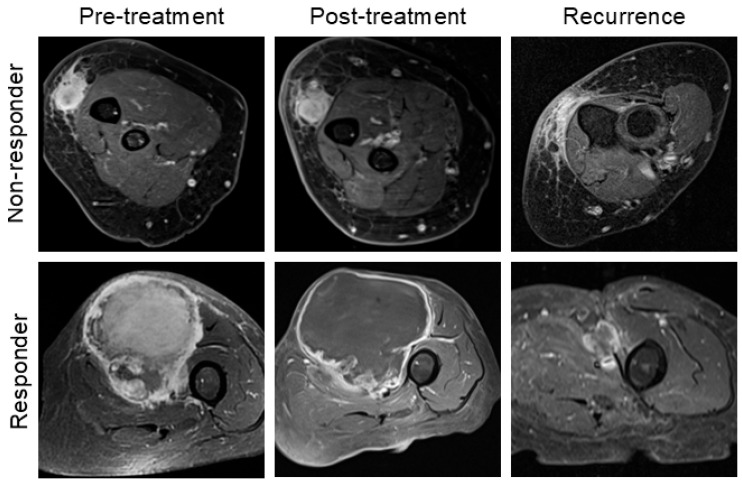
Upper row: A 71-year-old woman diagnosed with UPS (Grade II) in her right forearm. Upper row: Pre-treatment MRI shows a superficial, oval-shaped mass characterized by irregular margins and intense contrast-enhancement. After unsuccessful nCRT (viable cells 80%), size did not change significantly, nor the contrast-enhancement. Three years after surgical excision with margins of excision negative for tumor, an irregular area of nodular contrast-enhancement within the surgical bed was described at imaging. It was then confirmed histologically to be a recurrent UPS (Grade III), and the patient underwent re-excision. Lower row: An 84-year-old woman diagnosed with UPS (Grade III) in her left anterior thigh. Pre-treatment MRI shows a deep-seated round-shaped mass characterized by intense enhancement. After successful nCRT (viable cells <1%), the lesion did not show any enhancement areas but a slight increase in size likely due to hemorrhagic and necrotic content. The surgery was successful, with margins of resection negative for malignancy. Sixteen months after resection, a local recurrence was clinically suspected. After imaging and core-needle biopsy, final diagnosis of locally recurrent UPS (Grade III) was confirmed, and surgery was rescheduled.

Among patients treated with radiotherapy, 36% (ten out of twenty-eight) experienced recurrence, while 25% (two out of eight) of those treated with chemoradiotherapy recurred. We analyzed tumor size changes for both treatment groups, comparing patients who experienced recurrence to those who did not. Interestingly, responders with or without recurrence showed similar increase in tumor size, with no statistically significant difference. On the other hand, non-responders with recurrence exhibited a substantial reduction in all tumor dimensions compared to non-responders without recurrence. For non-responders this difference was statistically significant. Figure 4 illustrates the changes in main tumor diameters for responders and non-responders, comparing patients with and without recurrence. Detailed results are presented in Table 5. Both ANOVA and GLMM analyses (Figures S4–S6) independently confirmed these findings. Removal of three patients with positive margins did not alter these results (Figures S6). Due to the relatively small sample size, these findings should be interpreted cautiously. Figure 5 shows representative pre-treatment, post-treatment, and recurrence MRI scans for each response group.

### 3.4. Optimal Cutoffs for Response and Recurrence

**Figure 6:**
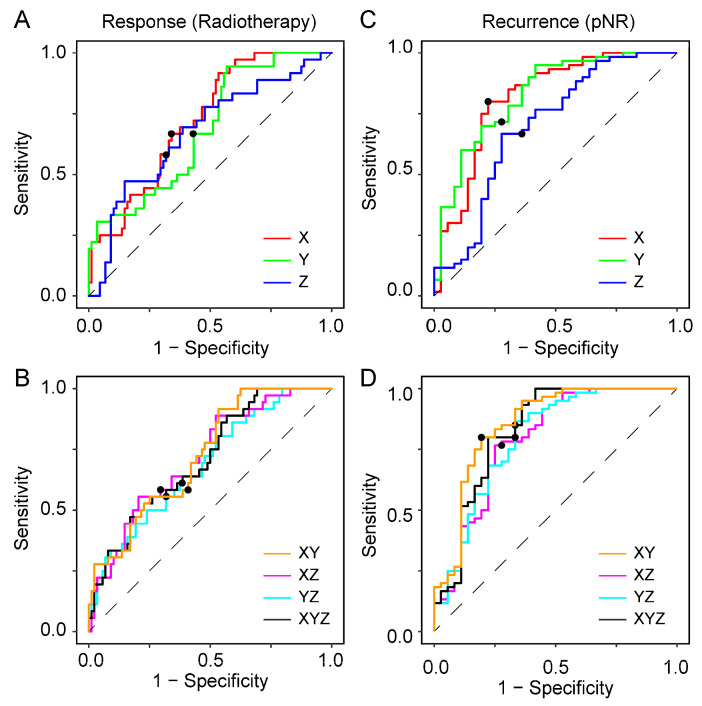
ROC curves illustrating the performance of different tumor size change cutoffs in predicting (**A**,**B**) response to radiotherapy and (**C**,**D**) recurrence among non-responders. The optimal cutoff (solid black dot) for each ROC curve was determined by maximizing the product of sensitivity and specificity.

Optimal cutoff analyses were performed to predict both pathological response to radiotherapy and local recurrence in non-responders (Figure 6). For predicting pathological response, the longest axial diameter (X) demonstrated the best overall performance, achieving a sensitivity of 0.69 and a specificity of 0.63 with an optimal cutoff of +19.6% (Table 6); smaller X changes corresponded to pNR. Figure S1 presents the results of optimal cutoff analyses for predicting pathological response to nCRT.

For predicting local recurrence in non-responders, the product of two perpendicular axial diameters (XY) showed the best performance, achieving a well-balanced sensitivity of 0.81 and a specificity of 0.78 at an optimal cutoff of +3.1%; smaller XY changes indicated potential recurrence. These cutoffs were subsequently used to assess the diagnostic performance of each model and compare it with the performance of RECIST 1.1. The results of this comparison are summarized in Table 7. Due to the small sample size, the statistical power of subgroup analyses is limited, and these preliminary results should be interpreted cautiously.

### 3.5. Diagnostic Performance of RECIST 1.1 and Optimized Cutoff Criteria

To evaluate the diagnostic performance of tumor size measurements and compare them with RECIST criteria, we adjusted the definition of progressive disease (PD) using thresholds determined through an optimal cutoff analysis. While the original RECIST 1.1 criteria defined PD as an increase in tumor size greater than 20%, our analysis led to the following adjusted definitions: for radiotherapy, PD was defined as a decrease in tumor size less than 19.6% (Table 6); for neoadjuvant chemoradiotherapy (nCRT), PD was defined as a decrease in tumor size less than 4% (Table S1). Table 7 compares the diagnostic accuracy of RECIST 1.1 and criteria based on the optimal cutoff computed for the longest axial diameter (X). RECIST 1.1 demonstrated the lowest accuracy and very low specificity, with a negative Kappa value indicating poor overall performance and substantial disagreement with pathological response. These findings are concordant with previously published results [5,25]. In contrast, the criteria based on the optimal cutoff exhibited high sensitivity and substantially improved accuracy compared to RECIST 1.1, with a positive Kappa value indicating some agreement. Both versions of the proposed criteria performed significantly better than RECIST 1.1, with a slight trade-off between sensitivity and overall accuracy.

## 4. Discussion

Rare cancers, comprising 27% of all diagnoses and 25% of cancer deaths, pose significant challenges, including delayed diagnosis, limited treatment options, and a lack of dedicated research. Soft tissue sarcomas (STSs) exemplify this, with limited treatment progress and outcomes over the past several decades [38]. Two primary factors contribute to this: a scarcity of clinical trials and an incomplete understanding of sarcoma biology [39]. Current clinical trials predominantly rely on tumor shrinkage, measured by RECIST 1.1 criteria [14], as the primary endpoint for treatment response. However, this approach is increasingly challenged by evidence, particularly in soft tissue sarcomas. Betgen et al. reported that only myxoid LPS responded to nCRT with tumor shrinkage, while other subtypes, including UPS, did not [40]. Other studies have shown heterogeneous response patterns, including a counterintuitive increase in tumor size, across various STS subtypes [9,10,11,13,21,22,41]. Given the limited research on UPS specifically, we focused on analyzing changes in tumor size in this most common STS subtype. Given the small sample size (n = 39) and limited statistical power (<0.4), the results of this study should be considered exploratory, and validation in larger, multi-institutional cohorts is necessary to confirm these findings and draw more definitive conclusions.

Our study demonstrated strong inter-reader agreement in assessing tumor size changes using diameter-based methods, a critical aspect of imaging biomarker development. Surprisingly, we observed an association between an increase in tumor size on MRI and improved pathological response. This finding highlights the importance of differentiating between true tumor growth and treatment-related changes in tumor size. While tumor size increase can indicate disease progression, it can also arise from treatment-induced effects like inflammation, hemorrhage, or necrosis. Radiation therapy, while primarily damaging DNA within the target volume, can also injure nearby blood vessel linings, leading to ischemia or hemorrhage, which can manifest as an increase or decrease in tumor size [42,43]. We previously reported significant correlations between tissue changes in UPS after radiotherapy [13]: necrosis and fibrosis were negatively correlated (−0.81), while volume change was positively correlated with necrosis (0.41) and negatively correlated with fibrosis (−0.44) in histology specimens. These relationships reveal distinct patterns in tumor response to radiotherapy. Necrosis and inflammation may represent early, volume-expanding responses, while fibrosis may be a later, more chronic response, potentially contributing to volume stabilization or contraction. The positive correlation between volume change and necrosis suggests that a larger volume increase is associated with more necrosis. Conversely, the negative correlation between volume change and fibrosis indicates that higher fibrosis is associated with a smaller volume increase (or even a decrease). This interplay between necrosis and fibrosis is likely dynamic, as suggested by the strong negative correlation between them and their respective correlations with volume change. Chemotherapy, which primarily exerts cytotoxic effects on cancer cells [6,16,24], typically leads to tumor shrinkage. The less pronounced size increase observed in the chemo-radiotherapy group suggests that chemotherapy might influence the balance between necrosis and fibrosis and cause lower degrees of inflammation and reduced radiation-induced edema, potentially through its anti-inflammatory properties. This could explain the smaller overall size increases observed in the chemoradiation group compared to radiotherapy alone. Importantly, the tumor size increase in responders suggests a form of pseudo-progression, indicating effective treatment response. However, while the term ’pseudo-progression’ is traditionally associated with immunotherapy-induced responses, characterized by an initial size increase followed by subsequent reduction, our findings suggest a distinct phenomenon in high-grade sarcomas like UPS [44]. In this context, ’pseudo-progression’ describes the observation of an initial size increase followed by a favorable pathological response, but the mechanism appears to differ significantly. In contrast, the minimal volume changes seen in non-responders reflect insufficient tumor cell death. Although stratifying patients by treatment regimen improved the distinction between responders and non-responders, the smaller chemoradiation group limited definitive statistical conclusions. Nevertheless, our analysis suggests a similar trend toward separation in both treatment groups, challenging the sole reliance on tumor shrinkage as an indicator of treatment efficacy in UPS patients. These findings suggest that diameter-based criteria optimized for UPS may offer a more accurate assessment of treatment response than RECIST 1.1, warranting further investigation. Clinically, high-grade sarcomas, such as UPS, are known to exhibit hemorrhagic changes following neoadjuvant therapy. This is further supported by clinical observations of rapidly enlarging masses that show no internal contrast enhancement and present with a heterogeneous MRI signal [5]. The variation in MRI signal arises from the differing stages of hemoglobin degradation, including metahemoglobin, intracellular oxyhemoglobin, intracellular deoxyhemoglobin, and extracellular methemoglobin, each of which displays distinct characteristics on imaging. For instance, subacute hemorrhage appears hyperintense on T1-weighted images, while acute hemorrhage is hypointense on T1. This observation challenges the conventional reliance on tumor shrinkage and underscores the need for refined imaging criteria that differentiate treatment-related changes, like hemorrhage, from true tumor progression in sarcomas. While an increase in size occurs in both responders and non-responders, as does hemorrhage, our findings demonstrate a significant difference in size increase between the two groups. Future studies should incorporate detailed analyses of non-enhancing areas on MRI, specifically focusing on signal characteristics indicative of hemorrhage, to validate this hypothesis and improve the accuracy of response assessment.

The primary aim of neoadjuvant therapy is to enhance local tumor control; however, consistent with the existing literature [17,42,43,45,46], this study found no statistically significant association between neoadjuvant therapy response and overall survival in UPS patients. While Kaplan–Meier analysis suggested a slight survival advantage for non-responders (Figure S7), Cox proportional hazards modeling revealed no significant difference between responders and non-responders (HR 1.27, 95% CI 0.40–4.00, *p* = 0.7). This uncertainty, reflected in the wide confidence intervals, suggests response status, as defined in this analysis, is not a significant predictor of survival. Similarly, recurrence data showed no significant association with survival (HR 1.42, 95% CI 0.38–5.23, *p* = 0.6). These findings are consistent with the understanding that overall survival in soft tissue sarcoma is likely more influenced by metastatic progression than local recurrence, and the wide confidence intervals likely reflect a limited sample size, underscoring the necessity for larger studies to validate these results and explore the impact of other clinical factors [47].

Assessing response to nCRT based on size changes provides valuable information, but predicting local recurrence after treatment remains a challenge. Several studies have correlated changes in size, including tumor volume, with the risk of local recurrence; however, these studies often used heterogeneous cohorts with subtypes showing conflicting responses to CRT [21,41]. Our single-subtype study of UPS revealed that responders experienced similar tumor size increases on MRI regardless of subsequent recurrence. While the percentage of viable tumor cells exhibited slight variation between patients with and without recurrence (6.3% ± 4% vs 3.5% ± 3.3%), this difference was not statistically significant, suggesting that changes in tumor size alone may be insufficient to identify patients at risk of recurrence following a favorable pathological response. Microscopic residual disease undetected after surgery could possibly explain recurrence in these cases. The percentage of viable tumor cells again did not differ significantly between non-responders with and without recurrence (53% ± 23% vs 45% ± 26%). Non-responders who did not experience recurrence showed similar size increases as responders. However, non-responders with eventual recurrence demonstrated minimal size changes or even a shrinkage. Notably, the difference in size change was statistically significant between responders and non-responders, allowing us to calculate optimal cutoff in tumor size changes (Table 5). While these results suggest potential for MRI size changes to predict recurrence in non-responders, it is crucial to recognize that pathological response may not always directly correlate with long-term disease-free survival. Further research is necessary to validate these findings and understand their long-term clinical implications. Moreover, incorporating advanced functional imaging techniques, such as diffusion-weighted MRI (DW-MRI), dynamic contrast-enhanced MRI (DCE-MRI), and contrast-enhanced susceptibility-weighted imaging (CE-SWI) [48,49,50,51], holds promise for a more comprehensive evaluation of treatment efficacy and prediction of local recurrence. DW-MRI provides insights into tumor cellularity by quantifying water diffusion within tissues. The apparent diffusion coefficient (ADC) can potentially distinguish between viable tumor and treatment-induced necrosis [51]. Similarly, DCE-MRI evaluates tumor vascularity by measuring contrast agent kinetics, which can differentiate active tumor regions from necrotic or fibrotic tissue after treatment. Contrast-enhanced subtraction-weighted imaging (CE-SWI) morphologic patterns strongly correlate with pathological response, can be easily recognized by radiologists without advanced post-processing software, and have the potential to outperform RECIST 1.1 [49]. Future research is essential to develop STS-specific prognostic and predictive biomarkers, involving larger sample sizes and incorporating advanced multiparametric imaging techniques (i.e., DCE-MRI, DWI, CE-SWI) to validate and expand these biomarkers, thereby deepening our understanding of tumor behavior after neoadjuvant treatment.

Small datasets are a major challenge in rare cancer research, often limiting the power of traditional statistical methods. Therefore, exploring more advanced statistical approaches is crucial. One promising strategy is to leverage multi-reader measurements. By incorporating multiple expert assessments of the same data, researchers can extract more information per patient, effectively increasing the sample size and improving the robustness of their analyses. Models like ANOVA and generalized linear mixed models (GLMMs) can be adapted for this purpose. Our findings were independently confirmed by both ANOVA and GLMM analyses, supporting this approach. This strategy, combined with advanced modeling techniques, can help maximize the insights gained from limited data and facilitate more meaningful conclusions in the study of rare cancers.

This study has several limitations. Its retrospective design, small sample size from a single institution (n = 39), and focus on tumor size without incorporating other multiparametric imaging markers limit the generalizability of the findings. Power analysis demonstrated limited statistical power (power < 0.4 to detect a medium effect size), as shown by wide confidence intervals of estimates, suggesting this study may be underpowered to detect true differences, particularly in subgroup analyses. Given the small sample size, we used both ANOVA and generalized linear mixed models (GLMMs) with multi-reader measurements, which independently confirmed our findings. However, this approach is not widely used and requires further validation. Variations in MRI timing, while reflecting real-world clinical practice guidelines, represent a key limitation. Specifically, the variable time interval between post-treatment imaging and surgery could affect the accuracy of tumor size measurements, particularly in cases where the tumor was increasing in size after therapy. A shorter interval might not allow sufficient time for the full treatment effect to manifest, potentially affecting the correlation between imaging findings and pathological response. This variability should be carefully considered in future larger studies. MRI measurements may also vary across institutions and protocols, highlighting the need for standardized imaging protocols to improve reproducibility. These factors also restricted the ability to conduct comprehensive subgroup analyses. The lack of external validation necessitates caution against applying these cutoffs in clinical practice without further investigation. Finally, relying solely on pathological response for stratification, without considering other clinical and prognostic factors, limited this study’s effectiveness in predicting recurrence.

## 5. Conclusions

In conclusion, this study highlights the challenges of predicting response and local recurrence in STSs. Subtype-specific focused analysis reveals the potential for accurate, even counterintuitive, imaging assessment compared to mixed-subtype studies. This study demonstrates that tumor size changes are significant predictors of pathological response and local recurrence in UPS. The marked differences in size changes between responders and non-responders underscore the potential of MRI to inform treatment decisions and facilitate risk stratification.

## Figures and Tables

**Table 1:** Patient demographics and clinical characteristics.

**Subject Count**	
Female	23 (59%)
Male	16 (41%)
Total	39
**Age**	
Female	64.5 ± 12.3 69.6 (50.5, 74.1)
Male	63.2 ± 16.1 67.5 (55.9, 74.3)
Average	63.7 ± 14.7 69.1 (54.8, 74.1)
**Grade**	
G2	11 (28%)
G3	28 (72%)
**Synchronous metastases**	
Metastases	1 patient (lung, brain)
**Tumor location**	
Upper extremity	5 (13%)
Lower extremity	32 (80%)
Retroperitoneum	1 (2.5%)
Trunk	1 (2.5%)

**Table 2:** Treatment, pathological details, and response to therapy. Abbreviations: RT—radiation therapy; PT—proton therapy; BL—pre-treatment baseline MRI; FU—post-treatment follow-up MRI. The interval between the BL and FU MRI scans is termed “Days between FU and BL imaging,” while the interval between the FU MRI and surgical resection is termed “Days between FU and Surgery.”

**Treatment**	
RT/PT, total dose and duration	50 Gy in 25 fractions (2 Gy/fx) in 5 weeks
Chemotherapy	Anthracycline-based (mostly doxorubicin) + ifosfamide
Days between FU and BL imaging	89.7 ± 26.1 91 (70, 108)
Days between FU and surgery	53.7 ± 87.2 21 (17, 36)
**Viable cell percentage**	
pR	4.1% ± 3.6%
pNR	48.3% ± 25.2%
**Pathological response**	
pR	15 (F:7, M:8)
pNR	24 (F:16, M:8)
**Surgical resection margins**	
Negative	29 (75%)
Positive	10 (25%)
**Local recurrence**	
Count	12 (F:9, M:3)
Grade	G2:6, G3:6
Treatment	Radiotherapy: 10 (36%) Chemotherapy: 2 (25%)
Years after surgery	3.67 ± 3.9 2.45 (1.3, 4.2)
**Metachronous metastases**	
pR	8 (54%)
pNR	11 (46)
Total	19
Lung	17 (89%)
Bone	5 (26%)
Abdominal	3 (16%)
Brain	3 (16%)

**Table 3:** Inter-reader agreement (Kendall W) for four readers and percentage change in tumor size for responders and non-responders, including the mean (±) standard deviation, median, and interquartile range. The mean difference (pR-pNR) between responders and non-responders, with 95% CI range and *p*-value assessed using Tukey’s HSD test.

%Change	Kendall’s W, *p*-Val.	pR n = 15	pNR n = 24	pR—pNR	*p*-Val.
X	0.88, 1.1 × 10^−12^	30 ± 35 15 (7, 39)	14 ± 31 9 (−6, 34)	16 (6, 27)	0.003
Y	0.87, 2.2 × 10^−12^	28 ± 42 8 (−0.3, 34)	13 ± 33 6 (−10, 30)	15 (3, 27)	0.015
Z	0.86, 3.3 × 10^−12^	22 ± 30 14 (2, 38)	12 ± 31 5 (−6, 26)	10 (0.4, 20)	0.04
X·Y	0.94, 3.8 × 10^−14^	77 ± 112 29 (7, 81)	36 ± 75 14 (−12, 65)	41 (12, 71)	0.006
X·Z	0.93, 8.9 × 10^−14^	67 ± 86 34 (7, 91)	35 ± 75 12 (−12, 59)	32 (6, 58)	0.016
Y·Z	0.92, 1.7 × 10^−13^	66 ± 97 19 (4, 86)	34 ± 77 11 (−17, 58)	32 (4, 60)	0.02
X·Y·Z	0.95, 2.5 × 10^−14^	143 ± 216 37 (8, 156)	72 ± 152 27 (−25, 105)	71 (13, 129)	0.017

**Table 4:** Percentage change in tumor size for responders and non-responders, including mean (± SD), median, and IQR, was computed for each group. Mean difference (pR-pNR) between responders and non-responders, with 95% CI range and *p*-value assessed using Tukey’s HSD test.

	Radiotherapy				Chemoradiation			
	pR n = 9	pNR n = 22	pR—pNR	*p*-Val.	pR n = 6	pNR n = 2	pR—pNR	*p*-Val.
X	44 ± 38 31 (14, 58)	17 ± 31 12 (−5, 34)	28 (15, 41)	4.9 × 10^−5^	8 ± 10 6 (2, 11)	−19 ± 14 −17 (−29, −8)	27 (18, 37)	1.6 × 10^−6^
Y	44 ± 46 24 (5, 91)	15 ± 33 9 (−6, 32)	28 (14, 43)	1.6 × 10^−4^	3 ± 15 −0.4 (−4, 6)	−15 ± 24 −15 (−34, 2)	19 (4, 33)	0.013
Z	34 ± 33 25 (9, 63)	14 ± 31 7 (−5, 27)	20 (7, 32)	0.002	5 ± 13 8 (−2, 12)	−9 ± 15 −7 (−13, −1)	14 (3, 26)	0.017
X·Y	120 ± 126 71 (27, 223)	42 ± 75 22 (−10, 66)	78 (42, 115)	3.9 × 10^−5^	13 ± 26 5 (−1, 9)	−29 ± 29 −26 (−59, −8)	42 (20, 65)	6.3 × 10^−4^
X·Z	102 ± 96 77 (24, 151)	40 ± 76 21 (−10, 62)	61 (29, 93)	2.6 × 10^−4^	14 ± 20 5 (−3, 16)	−26 ± 19 −26 (−39, −16)	41 (24, 57)	2 × 10^−4^
Y·Z	104 ± 109 60 (13, 209)	39 ± 78 15 (−13, 61)	64 (30, 99)	3.3 × 10^−4^	10 ± 26 7 (4, 22)	−23 ± 27 −28 (−44, −16)	33 (12, 55)	0.004
X·Y·Z	224 ± 246 116 (33, 443)	82 ± 155 35 (−17, 109)	143 (70, 215)	1.7 × 10^−4^	21 ± 38 8 (3, 26)	−35 ± 31 −43 (−59, −23)	56 (26, 86)	6.9 × 10^−4^

**Table 5:** Analysis of tumor size changes between responders and non-responders, categorized by patients with (Yes) and without (No) recurrence, including mean (±SD), median, and IQR. Mean difference (Yes/No), with 95% CI range and *p*-value assessed using Tukey’s HSD test.

	Recurrence in pR				Recurrence in pNR			
%Change	Yes n = 3	No n = 12	Yes/No	*p*-Val.	Yes n = 9	No n = 15	Yes—No	*p*-Val.
X	18 ± 9 15 (11, 24)	33 ± 38 15 (5, 45)	−15 (−38, 7)	0.18	−5 ± 26 −6 (−24, 1)	25 ± 28 16 (4, 42)	−31 (−42, −19)	8 × 10^−7^
Y	17 ± 14 19 (4, 25)	30 ± 46 6 (−2, 47)	−13 (−40, 14)	0.34	−9 ± 25 −13 (−23, 1)	26 ± 30 24 (−1, 47)	−35 (−47, −23)	1 × 10^−7^
Z	15 ± 13 17 (9, 23)	24 ± 33 13 (0, 52)	−9 (−28, 11)	0.37	−0.2 ± 27 −2 (−20, 11)	19 ± 30 12 (1, 27)	−20 (−32, −7)	0.002
X·Y	38 ± 24 34 (19, 60)	87 ± 123 25 (5, 97)	−49 (−120, 23)	0.18	−10 ± 48 −19 (−38, 0)	64 ± 75 48 (13, 74)	−74 (−101, −46)	9 × 10^−7^
X·Z	36 ± 18 38 (23, 48)	75 ± 95 23 (7, 108)	−39 (−94, 16)	0.16	−2 ± 53 −15 (−41, 5)	57 ± 78 35 (4, 68)	−58 (−87, −29)	1.6 × 10^−4^
Y·Z	36 ± 26 43 (16, 56)	74 ± 107 13 (3, 109)	−38 (−101, 24)	0.23	−5 ± 52 −20 (−43, 9)	57 ± 80 42 (3, 74)	−62 (−92, −32)	7.8 × 10^−5^
X∙Y∙Z	60 ± 35 67 (29, 236)	163 ± 236 34 (6, 223)	−103 (−241, 35)	0.14	−1 ± 86 −32 (−54, 7)	115 ± 166 64 (15, 118)	−116 (−175, −56)	2 × 10^−4^

**Table 6:** Optimal cutoffs (interquartile range), AUC, sensitivity, and specificity of different tumor size change measures in predicting response to radiotherapy and recurrence in non-responders.

	Response (Radiotherapy)				Recurrence (pNR)			
%Change	AUC	Optimal Cutoff, %	Sens.	Spec.	AUC	Optimal Cutoff, %	Sens.	Spec.
X	0.72	19.6 (15.7, 23.7)	0.69	0.63	0.82	0.7 (−1.1, 2.3)	0.80	0.76
Y	0.69	12.9 (6.3, 22.5)	0.65	0.55	0.83	0.8 (−3, 5.2)	0.75	0.74
Z	0.67	21.2 (16.8, 26.7)	0.59	0.68	0.70	2.9 (−2.7, 4.4)	0.70	0.67
X·Y	0.73	41.9 (30.1, 57.6)	0.64	0.60	0.85	3.1 (−2.1, 8.5)	0.81	0.78
X·Z	0.72	50.6 (37.4, 62.5)	0.62	0.68	0.79	1.3 (−2.5, 4.6)	0.79	0.71
Y·Z	0.69	47.2 (32.6, 61.1)	0.61	0.61	0.80	−3.1 (−7.3, 2.8)	0.81	0.69
X·Y·Z	0.71	87.3 (63, 111.4)	0.59	0.68	0.83	−2.9 (−11,3, 5.45)	0.83	0.71

**Table 7:** Diagnostic performance of RECIST 1.1 and criteria based on the optimal cutoff computed for the longest axial diameter (X) for patients treated with nCRT and radiotherapy only.

	**RECIST 1.1** **(nCRT)**	**RECIST 1.1** **(Radiotherapy)**	**Criteria for X** **(nCRT)**	**Criteria for X** **(Radiotherapy)**
Sensitivity	0.54	0.58	0.8	0.81
Specificity	0.34	0.19	0.48	0.43
Pos Pred Value	0.34	0.39	0.46	0.64
Neg Pred Value	0.53	0.33	0.82	0.64
Accuracy (95% CI)	0.42 (0.34, 0.50)	0.37 (0.29, 0.47)	0.6 (0.51, 0.67)	0.64 (0.55, 0.73)
Kappa	−0.11	−0.23	0.24	0.25

## Data Availability

Data are contained within the article and supplementary materials.

## Supplementary Materials

The following supporting information can be downloaded at: https://www.mdpi.com/article/10.3390/cancers17050830/s1, Figure S1: Percentage change in tumor size calculated using a generalized linear mixed-effects model (GLMM) for responders (pR) and non-responders (pNR) treated with radiotherapy and chemoradiation. Data are presented as mean ± standard deviation, with the mean difference (pR—pNR) between groups also shown; Figure S2: Percentage change in tumor size calculated using a generalized linear mixed-effects model (GLMM) for responders (pR) and non-responders (pNR) treated with radiotherapy. Data are presented as mean ± standard deviation, with the mean difference (pR—pNR) between groups also shown; Figure S3: Percentage change in tumor size calculated using a generalized linear mixed-effects model (GLMM) for responders (pR) and non-responders (pNR) treated with chemoradiation. Data are presented as mean ± standard deviation, with the mean difference (pR—pNR) between groups also shown; Figure S4: Percentage change in tumor size computed using generalized linear mixed-effects model (GLMM), for responders (pR) with and without recurrence (Yes/No). Data are presented as mean ± standard deviation, with the mean difference (pR—pNR) between groups also shown; Figure S5: Percentage change in tumor sizes computed using generalized linear mixed-effects model (GLMM), for non-responders (pNR) with and without recurrence (Yes/No). Data are presented as mean ± standard deviation, with the mean difference (pR—pNR) between groups also shown; Figure S6: Percentage change in tumor size for non-responders (pNR) who had negative surgical margins; three patients with positive margins were excluded. The analysis used a generalized linear mixed-effects model (GLMM) and compared groups with and without recurrence (Yes/No). The results are presented as mean change ± standard deviation, along with the mean difference between the groups (pR—pNR); Figure S7: Kaplan–Meier plot showing survival probabilities over time for a) non-responders (pNR) and responders (pR) and b) patients with and without recurrence. “+” symbols denote censored observations. Table S1: Optimal cutoffs (interquartile range), AUC, sensitivity, and specificity of different tumor size change measures in predicting response to nCRT.

## Abbreviations

The following abbreviations are used in this manuscript:

STS	soft tissue sarcoma
UPS	undifferentiated pleomorphic sarcoma
MRI	magnetic resonance imaging
DW-MRI	diffusion-weighted MRI
RECIST	Response Evaluation Criteria in Solid Tumors
nCRT	neoadjuvant chemoradiotherapy
pR	pathological responder
pNR	pathological non-responder
SD	standard deviation
IQR	interquartile range
GLMM	generalized linear mixed-effects model
