# Supplementary material for: Paradoxical Response to Neoadjuvant Therapy in Undifferentiated Pleomorphic Sarcoma: Increased Tumor Size on MRI Associated with Favorable Pathology
Source: Cancers (Basel). 2025 Feb 27;17(5):830. doi: 10.3390/cancers17050830 (PMC11899266; doi:10.3390/cancers17050830)
Supplement: Supplementary file 1 [file cancers-17-00830-s001.zip › cancers-3441444-Supplemental Figures S1-S6.pdf]

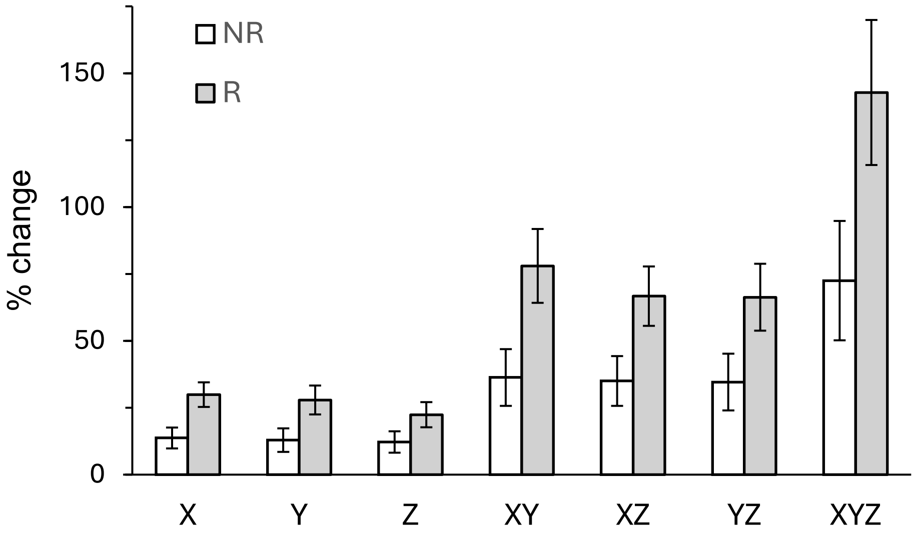

	R		NR		
	Mean	SD	Mean	SD	R - NR
**X**	29.9	4.6	13.7	3.9	16.2
**Y**	27.9	5.4	12.9	4.4	15
**Z**	22.4	4.7	12.2	4	10.2
**XY**	78	13.8	36.3	10.6	41.7
**XZ**	66.7	11.1	35	9.3	31.7
**YZ**	66.3	12.5	34.6	10.6	31.7
**XYZ**	142.8	27.1	72.5	22.3	70.3

Figure S1: Percentage change in tumor size calculated using a generalized linear mixed-effects model (GLMM) for responders (pR) and non-responders (pNR) treated with radiotherapy and chemoradiation. Data are presented as mean ± standard deviation, with the mean difference (pR - pNR) between groups also shown.

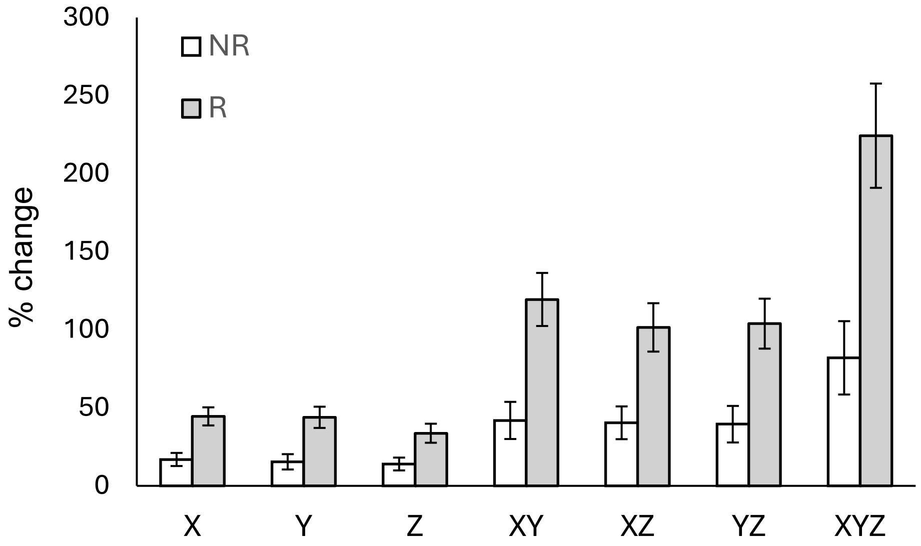

	R		NR		
	**Mean**	**SD**	**Mean**	**SD**	**R - NR**
**X**	16.9	4.2	44.5	5.8	27.6
**Y**	15.4	4.9	43.9	6.8	28.5
**Z**	14	4.1	33.7	6.1	19.7
**XY**	41.9	11.9	119.4	17	77.5
**XZ**	40.4	10.5	101.5	15.5	61.1
**YZ**	39.5	11.7	103.9	16	64.4
**XYZ**	82	23.5	224.3	33.4	142.3

Figure S2: Percentage change in tumor size calculated using a generalized linear mixed-effects model (GLMM) for responders (pR) and non-responders (pNR) treated with radiotherapy. Data are presented as mean ± standard deviation, with the mean difference (pR - pNR) between groups also shown.

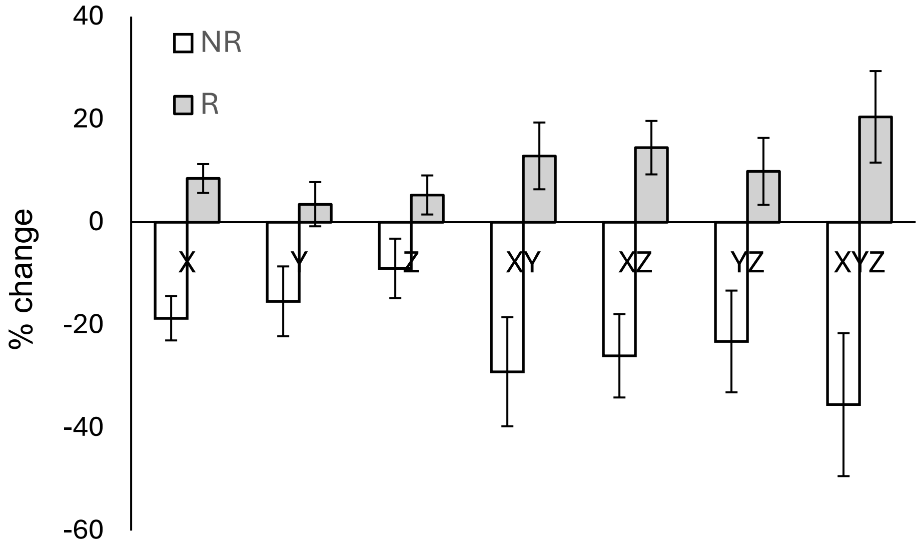

	R		NR		
	**Mean**	**SD**	**Mean**	**SD**	**R - NR**
**X**	-18.7	4.3	8.5	2.8	27.2
**Y**	-15.4	6.8	3.5	4.3	18.9
**Z**	-9	5.8	5.3	3.8	14.3
**XY**	-29.1	10.6	12.9	6.5	42
**XZ**	-26	8.1	14.5	5.2	40.5
**YZ**	-23.2	9.9	9.9	6.5	33.1
**XYZ**	-35.5	13.9	20.5	8.9	56

Figure S3: Percentage change in tumor size calculated using a generalized linear mixed-effects model (GLMM) for responders (pR) and non-responders (pNR) treated with chemoradiation. Data are presented as mean ± standard deviation, with the mean difference (pR - pNR) between groups also shown.

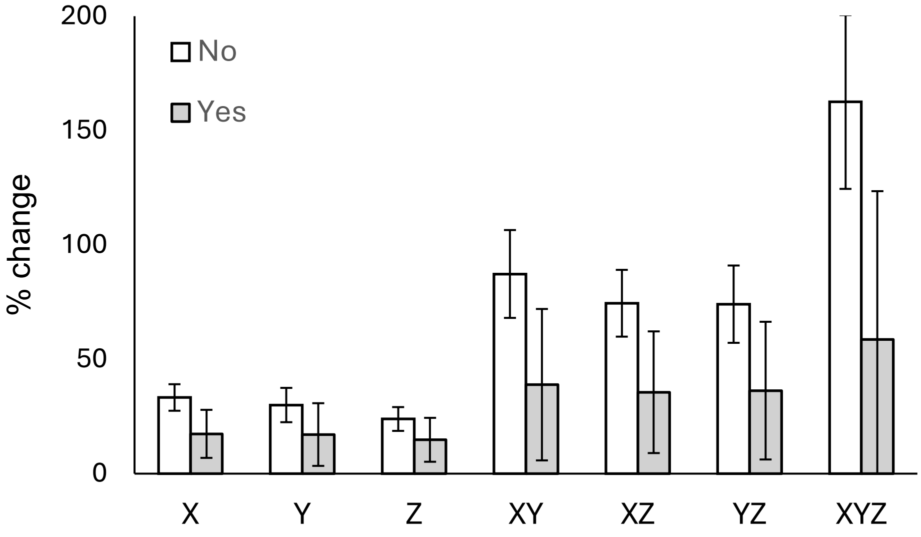

	No		Yes		
	**Mean**	**SD**	**Mean**	**SD**	**Yes - No**
**X**	33.3	5.8	17.4	10.5	-15.9
**Y**	30	7.5	17.1	13.7	-12.9
**Z**	23.9	5.2	14.8	9.6	-9.1
**XY**	87.3	19.2	38.9	33.1	-48.4
**XZ**	74.5	14.6	35.6	26.6	-38.9
**YZ**	74.1	16.9	36.3	30.1	-37.8
**XYZ**	162.5	38	58.6	64.9	-103.9

Figure S4: Percentage change in tumor size computed using generalized linear mixed-effects model (GLMM), for responders (pR) with and without recurrence (Yes/No).. Data are presented as mean ± standard deviation, with the mean difference (pR - pNR) between groups also shown.

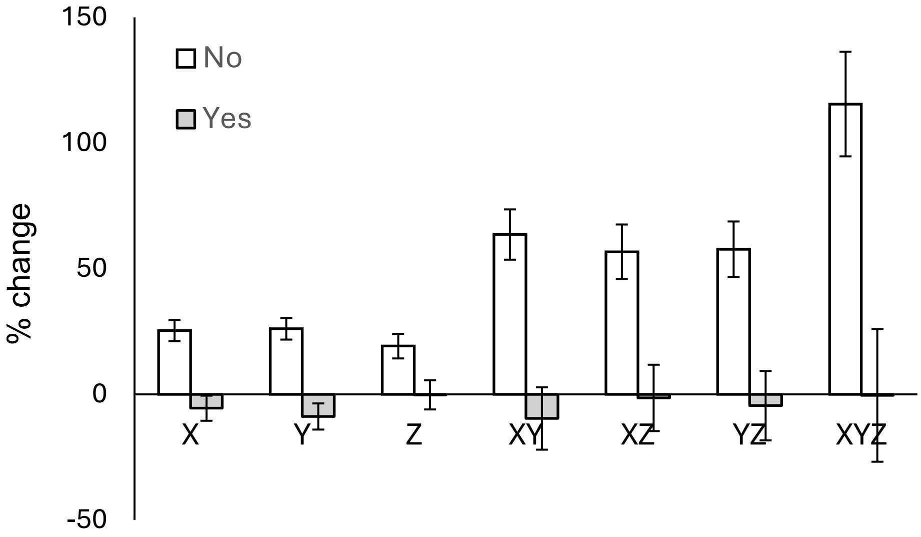

	No		Yes		
	**Mean**	**SD**	**Mean**	**SD**	**Yes - No**
**X**	25.4	4.2	-5.5	5	-30.9
**Y**	26.1	4.3	-8.8	5.2	-34.9
**Z**	19.2	4.9	-0.2	5.8	-19.4
**XY**	63.6	10	-9.6	12.4	-73.2
**XZ**	56.7	10.9	-1.4	13.2	-58.1
**YZ**	57.7	11.1	-4.5	13.8	-62.2
**XYZ**	115.5	20.8	-0.4	26.4	-115.9

Figure S5: Percentage change in tumor sizes computed using generalized linear mixed-effects model (GLMM), for non-responders (pNR) with and without recurrence (Yes/No).. Data are presented as mean ± standard deviation, with the mean difference (pR - pNR) between groups also shown.

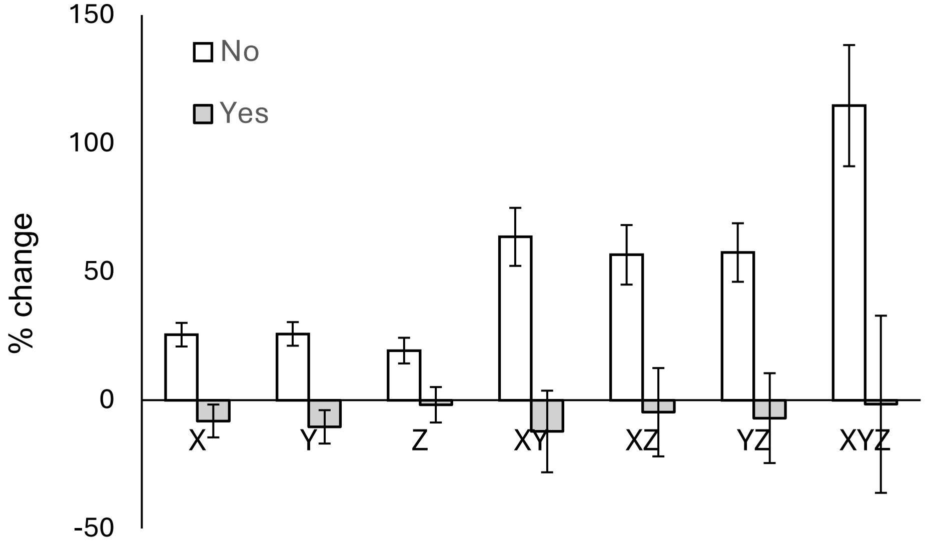

	No		Yes		
	**Mean**	**SD**	**Mean**	**SD**	**Yes - No**
**X**	25.5	4.6	-8.1	6.4	-33.6
**Y**	25.8	4.6	-10.4	6.5	-36.2
**Z**	19.3	5	-1.8	6.9	-21.1
**XY**	63.6	11.3	-12.2	15.9	-75.8
**XZ**	56.6	11.6	-4.7	17.2	-61.3
**YZ**	57.5	11.4	-7	17.5	-64.5
**XYZ**	114.7	23.6	-1.6	34.5	-116.3

Figure S6: Percentage change in tumor size for non-responders (pNR) who had negative surgical margins; three patients with positive margins were excluded. The analysis used a generalized linear mixed-effects model (GLMM) and compared groups with and without recurrence (Yes/No). The results are presented as mean change ± standard deviation, along with the mean difference between the groups (pR - pNR).
