# Supplementary material for: Paradoxical Response to Neoadjuvant Therapy in Undifferentiated Pleomorphic Sarcoma: Increased Tumor Size on MRI Associated with Favorable Pathology
Source: Cancers (Basel). 2025 Feb 27;17(5):830. doi: 10.3390/cancers17050830 (PMC11899266; doi:10.3390/cancers17050830)
Supplement: Supplementary file 1 [file cancers-17-00830-s001.zip › cancers-3441444-supplementary FigureS7.pdf]

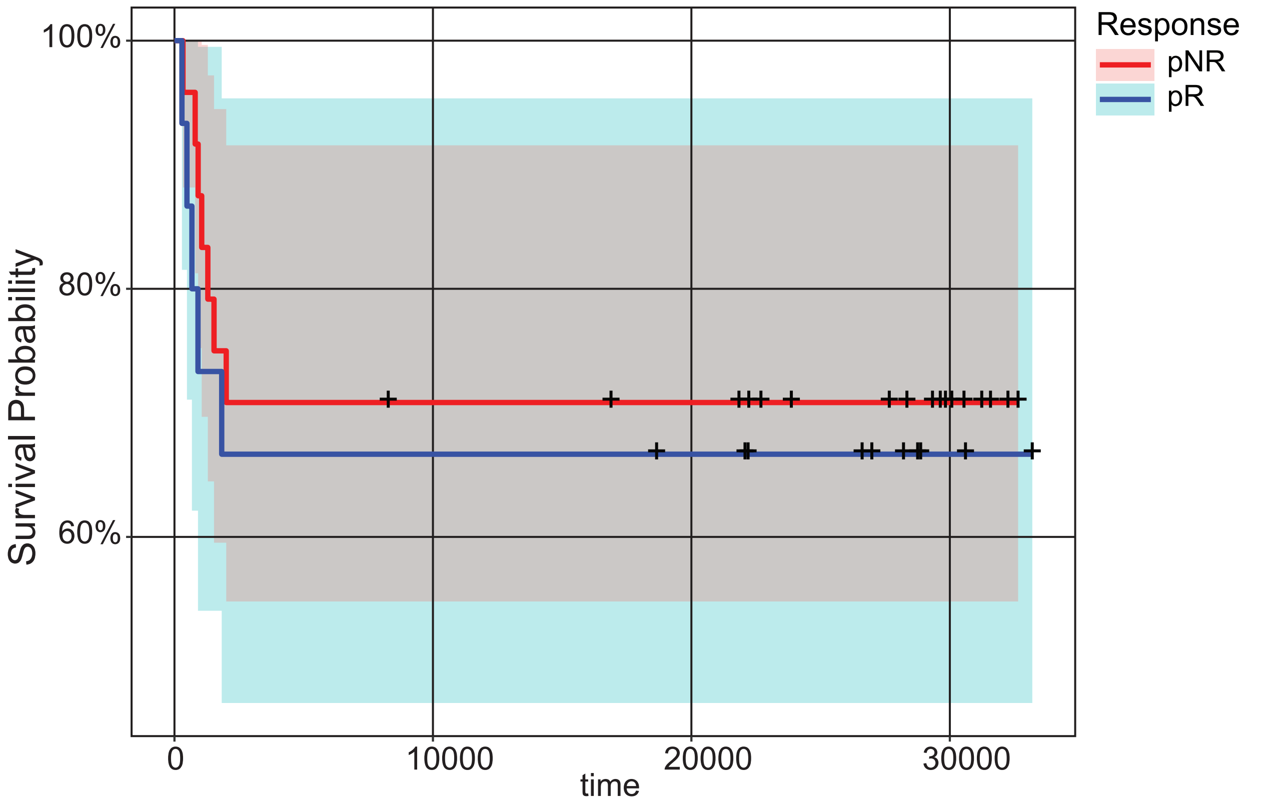

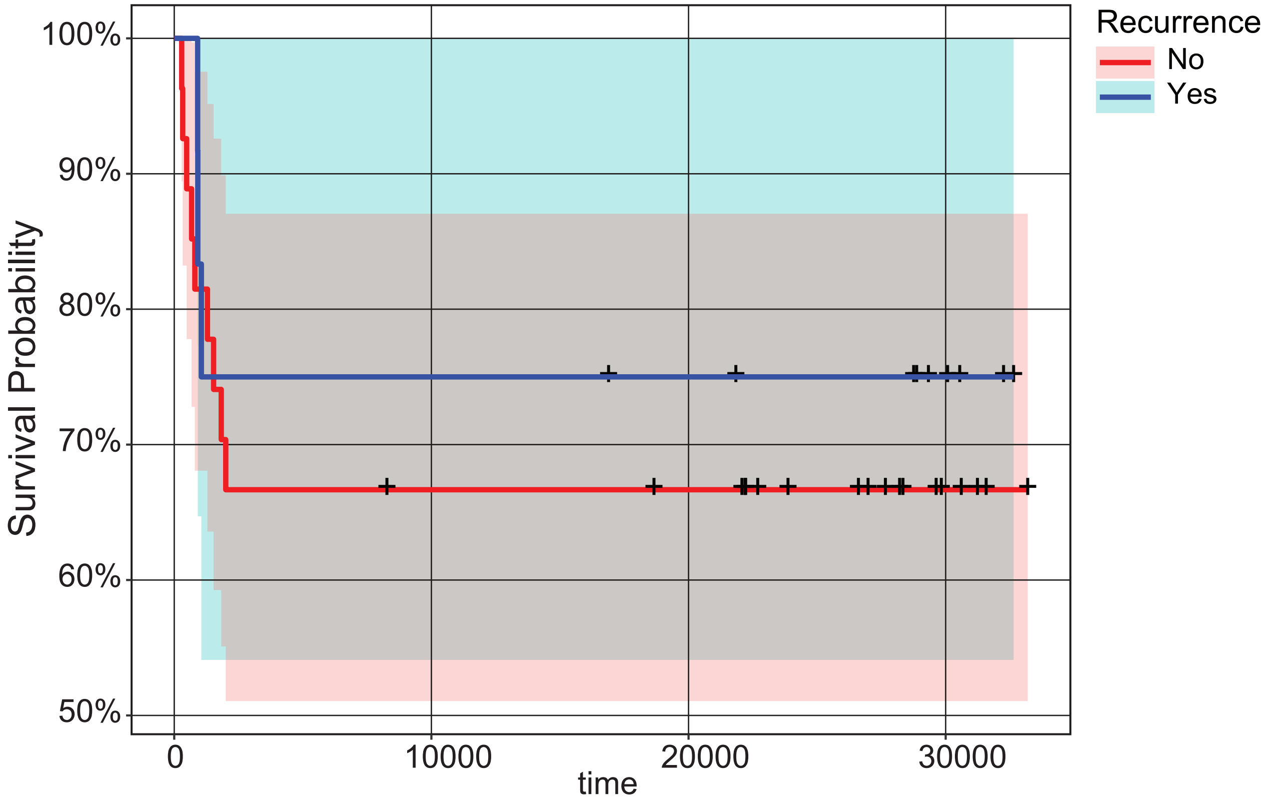

Figure S7: Kaplan-Meier plot showing survival probabilities over time for a) non-responders (pNR) and responders (pR) and b) patients with and without recurrence. "+" symbols denote censored observations.
