# Supplementary material for: Paradoxical Response to Neoadjuvant Therapy in Undifferentiated Pleomorphic Sarcoma: Increased Tumor Size on MRI Associated with Favorable Pathology
Source: Cancers (Basel). 2025 Feb 27;17(5):830. doi: 10.3390/cancers17050830 (PMC11899266; doi:10.3390/cancers17050830)
Supplement: Supplementary file 1 [file cancers-17-00830-s001.zip › cancers-3441444-Table S1.pdf]

Table S1: Optimal cutoffs (interquartile range), AUC, sensitivity, and specificity of different tumor size change measures in predicting response to nCRT.

	**Response (nCRT)**			
**%change**	**AUC**	**Optimal cutoff, %**	**Sens.**	**Spec.**
X	0.65	4.56 (2.9, 6.6)	0.82	0.46
Y	0.6	3.64 (0.3, 5.3)	0.67	0.48
Z	0.6	8.73 (7.4, 11.4)	0.67	0.54
X·Y	0.63	11.4 (4.3, 19.4)	0.68	0.43
X·Z	0.64	11.6 (6.6, 17.9)	0.73	0.45
Y·Z	0.61	8.77 (2.9, 19.9)	0.58	0.51
X·Y·Z	0.62	12.9 (3.3, 31.5)	0.63	0.47
